# Time is of the essence: an observational time-motion study of internal medicine residents while they are on duty

**Published:** 2017-06-30

**Authors:** Cameron W. Leafloor, Erin Yiran Liu, Catherine C. Code, Heather A. Lochnan, Erin Keely, Deanna M. Rothwell, Alan J. Forster, Allen R. Huang

**Affiliations:** 1 Faculty of Medicine, University of Ottawa, Ontario, Canada; 2 Quality and Performance Measurement, Ottawa Hospital, Ontario, Canada; 3 Division of General Internal Medicine, The Ottawa Hospital, Ontario, Canada; 4 Division of Endocrinology and Metabolism, The Ottawa Hospital, Ontario, Canada; 5 The Ottawa Hospital Research Institute, The Ottawa Hospital, Ontario, Canada; 6 Division of Geriatric Medicine, The Ottawa Hospital, Ontario, Canada

## Abstract

**Background:**

The effects of changes to resident physician duty hours need to be measureable. This time-motion study was done to record internal medicine residents’ workflow while on duty and to determine the feasibility of capturing detailed data using a mobile electronic tool.

**Methods:**

Junior and senior residents were shadowed by a single observer during six-hour blocks of time, covering all seven days. Activities were recorded in real-time. Eighty-nine activities grouped into nine categories were determined *a priori*.

**Results:**

A total of 17,714 events were recorded, encompassing 516 hours of observation. Time was apportioned in the following categories: Direct Patient Care (22%), Communication (19%), Personal tasks (15%), Documentation (14%), Education (13%), Indirect care (11%), Transit (6%), Administration (0.6%), and Non-physician tasks (0.4%). Nineteen percent of the education time was spent in self-directed learning activities. Only 9% of the total on duty time was spent in the presence of patients. Sixty-five percent of communication time was devoted to information transfer. A total of 968 interruptions were recorded which took on average 93.5 seconds each to service.

**Conclusion:**

Detailed recording of residents’ workflow is feasible and can now lead to the measurement of the effects of future changes to residency training. Education activities accounted for 13% of on-duty time.

## Introduction

Prolonged on call shifts and long duty hours are linked to concerns for patient safety, quality of care as well as concerns for the health and safety of resident trainees who may be working in excess of 24-hours without relief.^1^,^2^ The 2003 European Working Time Directive,^3^ the mandate as described by the U.S. Accreditation Council for Graduate Medical Education (ACGME) 2017 regulations,^4^ and the Institute of Medicine 2009 report “Resident Duty Hours: Enhancing Sleep, Supervision, and Safety”^5^ have influenced the implementation of restrictions on resident duty hours. In addition, academic health centers have implemented various on-call strategies such as specific night shift relief to help ease resident on call duties or decrease length of consecutive duty hours. Recent publications^6^–^8^ have highlighted the ongoing debate concerning the duration of duty hours, its potential harms and benefits.^9^–^11^

As discussions around the world continue in consideration of further changes to resident physician duty hours, it is important to have an improved description of workflow in order to measure the impact of new systems of workflow and determine how they will affect patient care, resident education, and resident work-life balance. Having an easy to use and reliable tool for workflow determinations is essential to study the effects of future changes to resident duty hours.

Time-motion studies have been used both in industry^12^ and in hospitals^13^–^16^ to provide an objective method to record time for completed tasks. A previous systematic review of time-motion studies of internal medicine residents identified eight studies which lacked detail in categorization or definition of how time was spent with limited ability to detect trends related to restrictions on duty hours.^17^ Residents spent the highest percentage of time in patient care ranging from 27–52% though extrapolating to actual number of hours was not possible with the published data. The percentage time spent on education was modest and decreased minimally after implementation of duty hours restrictions. The meta-analysis demonstrated the paucity of information and the need for detailed moment to moment description of resident work flow.^17^

The purpose of this study was to determine the exact amount of time Internal Medicine residents spend on specifically defined tasks while on duty on an inpatient clinical teaching unit (CTU) in an academic hospital. The secondary goal of this project was to test the feasibility of an electronic data capture tool to quantify in minutes how residents spend their time. By understanding and measuring resident workflow future changes to training programs and scheduled activities can be designed to optimize resident efficiency, improve physician-patient interactions, and potentially enhance patient care and safety.

## Methods

This study was performed at The Ottawa Hospital in Ottawa, Ontario, Canada. The Ottawa Hospital is an academic health center which serves a total population of 1.25M. The Ottawa Hospital has two sites: the General Campus (549 beds) and the Civic Campus (460 beds). All participants were enrolled in the core Internal Medicine residency training program.

This was an observational time-motion study of Internal Medicine residents, including junior (postgraduate year PGY-1), and senior (PGY-2 and 3) residents during their rotation on the internal medicine CTU.

**Figure 1:**
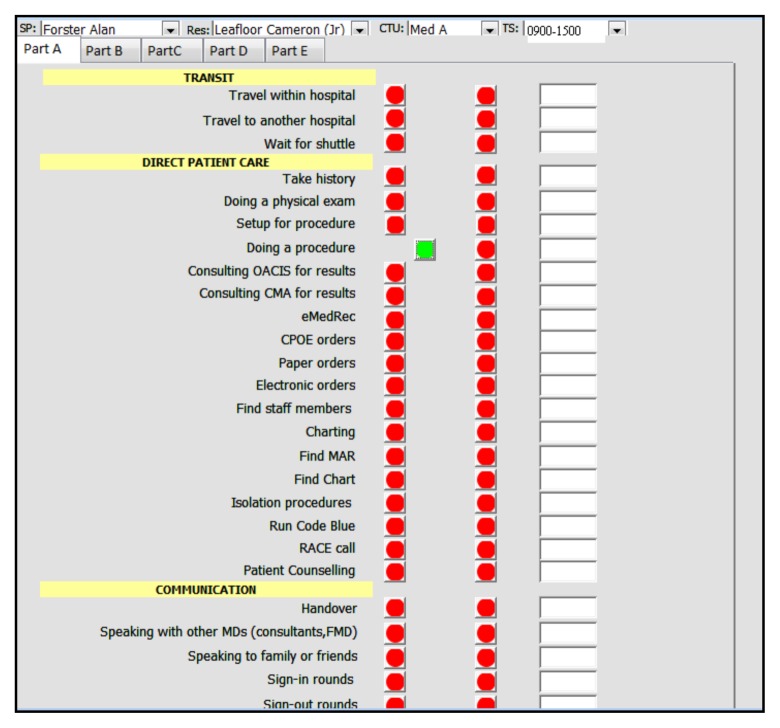
Screenshot of the data capture tool application

**Figure 2:**
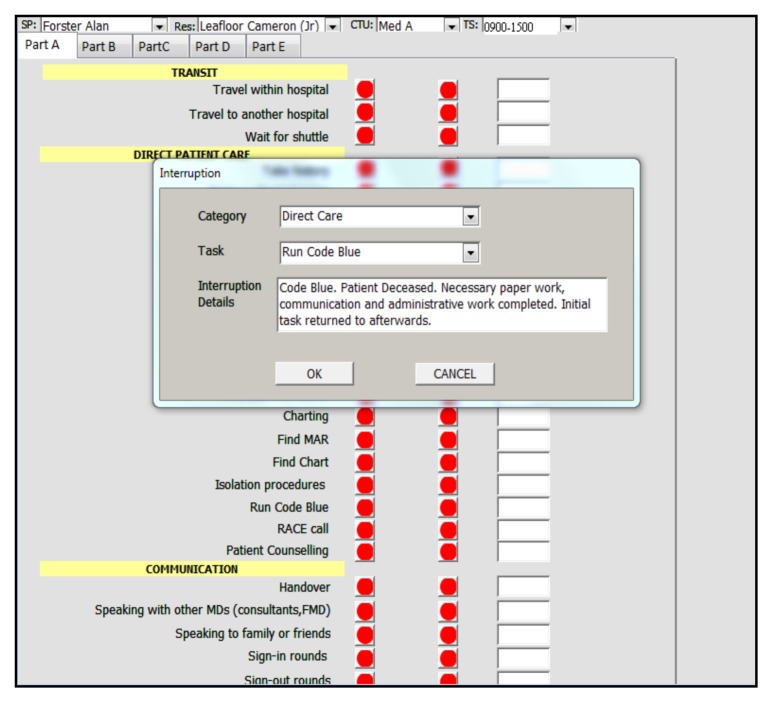
Screenshot of the data capture tool recording a task interruption

All residents in the core Internal Medicine residency training program were invited to participate. Participants signed informed consent. The list of tasks and their categories was determined *a priori* by a focus group of 27 participants of which there were eleven PGY-1 residents, seven PGY-2, four PGY-3 and five attending staff. The initial list of tasks and categories were derived from the results of a systematic review.^17^ This list was then expanded, refined and finalized via a Delphi process through three iterations.^18^ The final list of nine categories (Direct patient care, Indirect patient care, Education, Communication, Administrative tasks, Documentation, Personal Tasks, Transit, and Non-physician Tasks) containing 89 tasks are listed in Appendix A. The data collection grid covered two resident types, 7-days/week, 24-hours/day (see Appendix B). Both campuses of The Ottawa Hospital and six Internal Medicine CTUs participated. Residents were shadowed by a single observer (CL), who was a health sciences undergraduate student. The 56 separate observation blocks were completed in a random sequence, since the single observer could not feasibly do shadowing activities for more than up to 12-hours at a time. The observer used an application running on a wireless mobile device to record all tasks and their duration. The application was essentially a set of data entry forms designed using Microsoft Access 2003 (Microsoft Canada, 1950 Meadowvale Blvd, Mississauga, ON, L5N 8L9). Brief technical details concerning the mobile application are included in Appendix C. At the start of each observation block a comment could be recorded to provide additional context to the observations, e.g., “Emergency Room pressure is high today.” Additionally, the application was able to record interruptions, including their duration and details. Figure 1 shows a screenshot of the mobile application and Figure 2 shows a screenshot of data entry concerning a task interruption. The observer tapped the button next to the corresponding task to be recorded, which captured the date and time of the start of that activity. Tapping the green button (which indicated that an activity was in progress) captured the date and time of the end of that activity. A comment could be entered for each activity recorded. Each activity had to be completed before recording a new one, except in the case of an interruption. Simple descriptive statistics of the data were done using SAS version 9.3 (Cary, N.C., USA). The study protocol was approved by the Ottawa Health Sciences Network Research Ethics Board.

The single observer (CL) underwent training, including the use of the mobile data collection tool, understanding what a typical resident’s day entailed and piloting the entire system through five trial run observation blocks which covered both day and night shifts. The observer was instructed to limit interactions with residents as much as possible, only asking if a clarification about the current task (e.g., a computer being used for which purpose) was needed. The study took place between June 19 and August 20, 2013.

## Results

**Figure 3:**
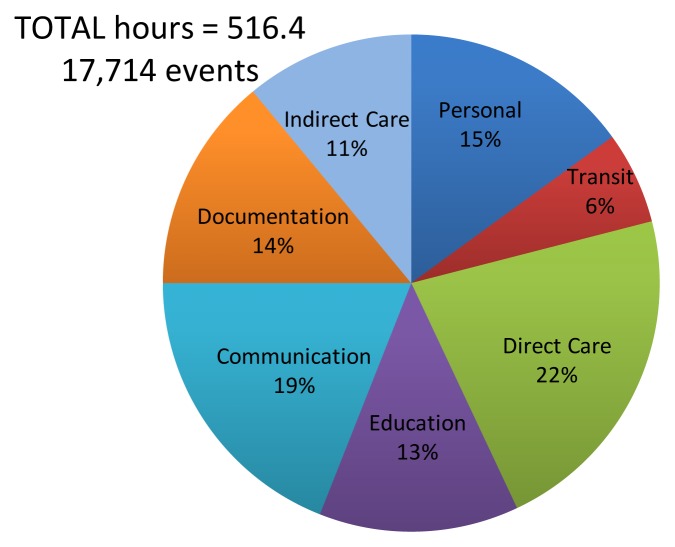
Total hours and percentages of time spent in each of the categories of tasks

Twenty-six of 80 eligible residents (32.5%) in the core Internal Medicine residency program consented to participate. Thirty-six of these residents would have been scheduled for CTU rotations during the study period. Twenty residents were shadowed over 61 blocks of time (which included the five trial observation blocks), recording 17,726 tasks and 968 interruptions. Twelve records were excluded because of missing data, resulting in a pool of 17,714 tasks. A total of 516 hours were recorded, which includes multi-tasking time. Observation records were nearly evenly distributed: junior residents (51%), senior residents (49%), Civic site (47%) and the General site (53%). Figure 3 shows the total hours and percentages of time spent in each of the categories of tasks. Direct Care activities (111.6 hours, 22%) took up the largest amount of time, followed by Communication (98.7 hours, 19%); Personal (76 hours, 15%); Documentation (69 hours, 1%); Education (68.2 hours, 13%); Indirect care (57.7 hours, 11%); Transit (30.2 hours, 6%); Administrative (3 hours, 0.6%); and Non-Physician tasks (1.8 hours, 0.4%). Table 1 shows what activities filled most of the times spent in the different categories of tasks. Some activities, for example in the education category, happened less often and took more time, such as didactic large group and bedside teaching. The majority of Direct Care activities did not require physicians to be at the bed side and involved using computer applications. During their entire on duty time residents spent 8.8% of their time in the physical presence of their patients, of which 42.1% of this time was spent in patient counseling activities.

### Interruptions

An interruption was defined as the recording of an event which caused the resident to switch focus from a current activity to complete a different one. For example, if a resident was paged and stopped the current activity to answer their page, this was considered an interruption. However, if the resident completed the current activity and then answered their page, this was not considered an interruption. The paging event was no longer interrupting the completion of an activity but became the next task on the resident’s to-do list. The study recorded 968 interruptions, for a total of 95.76 hours with an average duration of 93.5±198.82 seconds. The most common interruptions were caused by talking to attending staff in person (50.1%) and answering pages (20.7%). Since the time recorded to the original activity that was interrupted includes the time needed to service the interruption, the total observation time of 516 hours exceeded the elapsed total real time of 366 hours (61 blocks x six-hours). The timer for the original task, which was interrupted, was continued since we wished to document whether residents actually returned and completed those tasks. There were a total of 36 tasks which were interrupted and were never resumed to completion (e.g. cardiac arrest code interrupting a patient counselling activity).

## Discussion

The time-motion data capture by a single observer using a mobile device application was easy to use and produced detailed, minute-to-minute information. The application enabled the recording of task interruptions and unlike using wearable video recording devices a live observer could ask for clarification (e.g., whether the computer use being observed was associated with patient care or education). In comparison to other time-motion studies as reported in a systematic review by Leafloor et al.^17^ this study yielded superior data than those which used paper and stopwatches, relied on resident reporting or recall, or had limited number of observation events or time. Since the list of tasks and categories were determined by consensus *a priori* it was easy to record the tasks in real time. The entire data capture system was developed and implemented with readily available and inexpensive software. The use of a mobile tablet device and wireless communications made data entry easy and allowed the capture of timestamps in real time. Security, privacy, and confidentiality were maintained since the mobile device served only as a user interface and stored no data. The entire solution, including hardware and software were affordable and easily available. The pre-requisite for transferability of this technology elsewhere is the reliability and quality of service of the in-hospital wireless communications infrastructure. Other studies reported similar average proportion of time devoted to Direct Care of patients (41.81%), Communication (18.19%), Personal/other (19.59%), and Education (13.03%).^17^

### Direct patient care

A trend to decreased time physicians spend at the bedside has been seen since the first restrictions in duty hours were implemented.^13^,^14^,^16^,^17^,^19^–^21^ The change is striking when comparing the 1990 study by Nerenz, who found 17% of the residents’ time (4.08 hours of a 24-hour call shift) was spent in direct contact with patients^20^ compared with 9.21% (2.21 hours of a 24-hour call shift) found by Block.^14^ One possible factor is the increasing availability of electronic clinical data which has replaced the need to question patients about their medical histories. In our study, 50.7% of time spent (see Table 1) in direct patient care activities involved the access of electronic data which did not require direct patient contact. However less time spent with patients has been associated with a decrease in patient satisfaction.^22^–^24^ To offset the finding that residents spent a significant proportion of time looking at electronic data, they also spent 17.3% of time in the direct patient care category talking to and counselling patients.

### Communication

Duty hour restrictions seem to be associated with an increase in the frequency and total duration of communication activities. This study found that 65.0% (17.0% sign-in rounds, 4.2% sign-out rounds, 41.6% talking to hospital staff) of time was spent in information transfer activities, compared to 16.5% of time reported in studies before 2003 and 22.3% of time reported in studies afterwards.^17^ These exchanges of clinical information including handover are an essential aspect of healthcare delivery, and if handover is not standardized there is a potential for information gaps leading to possible adverse events.^25^,^26^ Interruptions during handovers can potentially disrupt efficient information transfer and an analysis of our dataset into this issue will be the subject of a separate study.

### Education

Meeting resident educational objectives is an important aspect in the management of workflow. Our study found the proportion of time the residents spent in educational activities (13.2%) during their entire on-duty time was similar to that found in the systematic review (average 13%)^17^ and the 15% reported by Fletcher.^16^ A significant 42.8% of events taking 18.8% of time within the Education category were self-directed, comprising of information searches using medical databases, internet searches, and literature searches. Previous research had also found an increasing portion of self-directed learning activities.^13^,^16^,^19^,^20^ The largest portion (25.9%) of time was spent with senior residents reviewing new cases with junior residents. The detailed case review, either one-on-one or during morning report can fulfill several objectives: provide a learning experience, implement quality control, and enhance patient flow management. Our data recorded only four instances of bedside rounding events. This low number may reflect the non-consecutive observation blocks that missed rounds which were planned around a particular attending physician’s schedule, an idiosyncrasy related to when the study was done (at the beginning of the academic year) or other unidentified confounders. Further clarification will be explored since bedside rounding has been at the core of medical teaching and has been received positively by learners.^27^–^29^ Across multiple studies of medicine and surgery programs there has been a decrease in educational opportunities^30^,^31^ with duty hour changes. Prior studies have shown that while the number of hours residents were able to sleep has increased and perceived fatigue decreased, the quantity and quality of educational opportunities for residents as well as the quality and continuity of care they provided decreased significantly.^21^,^32^ It is important to note that activities were categorized as Education by the residents themselves and for the most part did not consider hours spent in situational, work based or experiential learning environments. Re-categorizing some or a portion of these events may alter the percentage of time spent in learning.

### Personal

Time spent in personal activities (14.7%) was similar to the average of 13.7% found by Leafloor,^17^ of which half was spent in the on-call room presumably providing an opportunity to sleep. Although there were un-recorded events during the time residents were in the on-call room (e.g. answering pages), our hospital has implemented a rule that junior residents are not to be called between 03:00–08:00 for a new consult or admission that requires an in-person visit. A meta-analysis of the impact of reduced duty hours in surgical residents found an improvement in resident fatigue levels and general well-being.^30^ A 2006 study also reported that surgical residents spent more time having lunch after the 2003 duty hour changes and reported a decrease in “emotional burnout.”^31^ These findings suggest that duty hour changes do support a better life-work balance for residents.

### Strengths of the study

To our knowledge, this study reports the results of the most comprehensive and complete time-motion analysis of internal medicine residents to date. The categories and tasks were selected *a priori* and recorded consistently by a single observer. Time was automatically recorded and the wireless mobile technology ensured complete and reliable real-time data capture.

### Limitations

Despite precautions taken to minimize interactions between the observer and the residents, the mere presence of an observer may have changed their behavior. The Hawthorne effect^33^ may have caused residents who were observed to avoid doing non-work related activities while being shadowed. Although activities of a personal nature were indeed recorded (conversations, emails, social media, etc.) we would not be able to measure the suppressive effects of having a shadow observer. Other confounders include: resident factors, hospital environment factors, time block factors, and day of week effects. Residents who did not consent or were not observed may be different. This study was done during the beginning of the academic year which may be different. The use of a single observer versus multiple observers may have introduced measurement bias due to a single observer’s interpretation of witnessed events. The observer may misclassify some observed events. However, clarification and validation of events were done in real-time if needed. Personal time may have embedded events such as answering pages and issuing orders while residents were in their on-call room. Some communication activities, for example, talking to attending staff by phone, may have embedded learning points and some of that time should contribute to education time. The physical layouts of the Civic and General campuses are different. The observation blocks were not all done in consecutive sequence. Different attending staff physicians may have different scheduled teaching or case review events. A possible “gold standard” for time-motion study by analyzing the continuous recording of a subject while wearing a personal video recording device (e.g. GoPro) was not considered because of the significant privacy issues involved.

### Conclusions

Time-motion observation of residents while they are on duty on an internal medicine clinical teaching unit using a mobile data capture tool can generate comprehensive, complete, and detailed records. Understanding how time is spent while residents are on duty can assist discussions with residency program directors, hospital management, quality and safety directorates and resident physicians. Different work patterns can be designed, tried and measured for their effects on patient care and satisfaction, resident learning experiences and resident well-being. Further research studies employing different methodologies are needed to clarify the effects of some of the above-identified limitations. Authorship: All authors contributed to the design of the study. CL and AH were involved with the design of the mobile data collection tool and data collection process. EL and DMR were involved in the data analysis. All authors were involved in the interpretation of the results and writing of the manuscript. All authors read and approved the final text.

## Figures and Tables

**Table 1:** Listing of tasks (17,714 total events, 516.35 hours) in each category which comprise the majority of time spent. Percentages of the number of tasks and of time spent within each category are reported. The percentages of the number of events and of time spent in each category across the entire data set is also reported.

Task	% number	% time

**Total time in presence of patients**	**8.9**	**8.8**

**Direct Care (4445 events, 111.6 h)**	**25.1**	**21.6**

Consult electronic medical records	30.7	30.4
Patient Counselling a	18.1	17.3
Consult mobile electronic results	13.8	16.8
Paper order	15.9	12.0
Take history	8.3	8.1
Doing a physical exam	7.5	7.6
(Using any electronic systems)	47.2	50.7

**Communication (3587 events, 98.7 h)**	**20.2**	**19.1**

Communication with hospital staff	65.6	41.6
Sign-in rounds	0.7	17.0
Speaking to family or friends	9.9	11.1
Phone call	8.8	8.9
Wait for page	2.9	7.3
Answer Page	3.7	5.2
Sign-out rounds	0.3	4.2
(Information transfer activities)	67.5	65.0

**Personal (1207 events, 75.96 h)**	**6.8**	**14.7**

In Call Room	2.5	50.5
Eating	9.7	18.3
Personal conversation	53.4	15.6
Other personal	30.5	12.4
Personal emails, social media	3.3	3.0

**Documentation (944 events, 69.01 h)**	**5.3**	**13.4**

Consultation	36.3	49.3
Progress notes	33.2	25.4
Admission notes	14.0	11.7
Discharge summaries	9.4	11.1

**Education (1053 events, 68.2 h)**	**5.9**	**13.2**

Double checking Jr tasks	19.2	25.9
Being reviewed by Senior or	11.5	16.8
Attending	1.5	15.6
Didactic teaching: Large group^b^	20.6	7.5
Information searching [UPtoDate]^c^	15.6	6.0
Internet resources	8.0	5.4
Being taught	6.6	5.2
Literature search	10.0	2.5
Teaching others one-on-one	1.4	2.0
Teaching others	0.2	1.0
Didactic teaching: small group b	0.4	1.0
Beside rounding (self-directed learning activities)	42.8	18.8

**Indirect Care (4015 events, 57.72 h)**	**22.7**	**11.2**

Reading Chart	55.6	73.5
Updating patient lists	19.7	18.4
Finding Medication Admin Record	2.0	0.9
Wash hands	13.0	2.8
Finding chart	7.8	2.8

**Transit (2146 events, 30.2 h)**	**12.1**	**5.8**
**Administrative (104 events, 3.04 h)**	**0.5**	**0.6**
**Non-MD tasks (213 events, 1.8 h)**	**1.2**	**0.4**

Travel within hospital (Transit)	99.5	91.3
Respond to staff emails (Admin)	77.9	85.7
Search for supplies (Non-MD)	96.2	93.4

a Patient Counseling: communicating with patients casually, discussing impression/plans or goals of care with patient, consoling patient.

b didactic teaching, large group=12 or more learners, small group=less than 12

c UptoDate is a medical knowledge database (www.uptodate.com)

**Figure:**
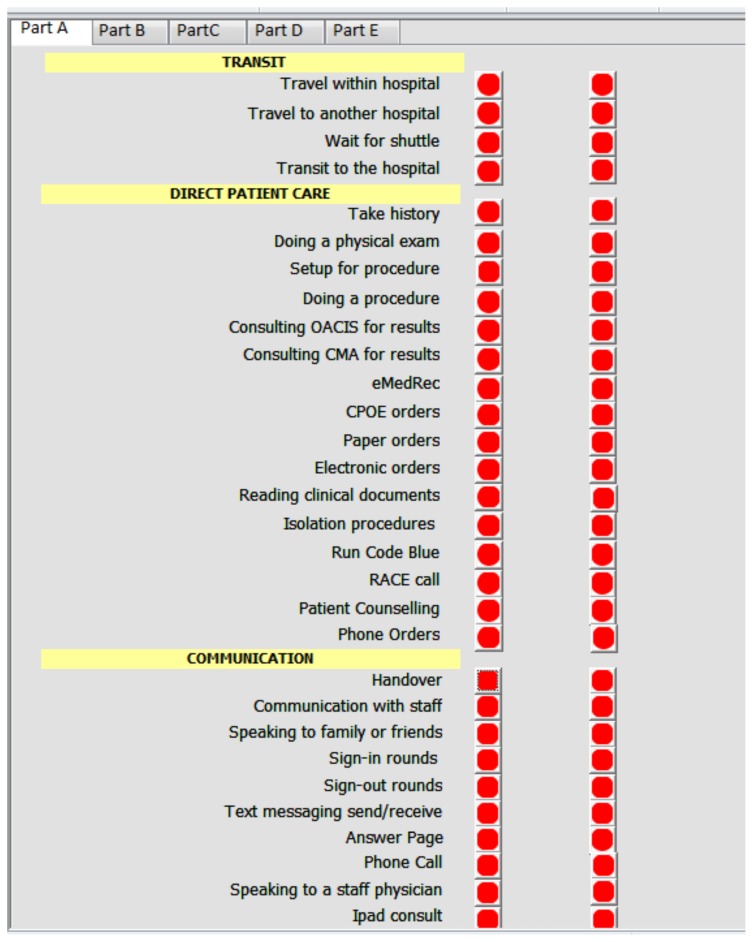
Form Layout Part A

**Figure:**
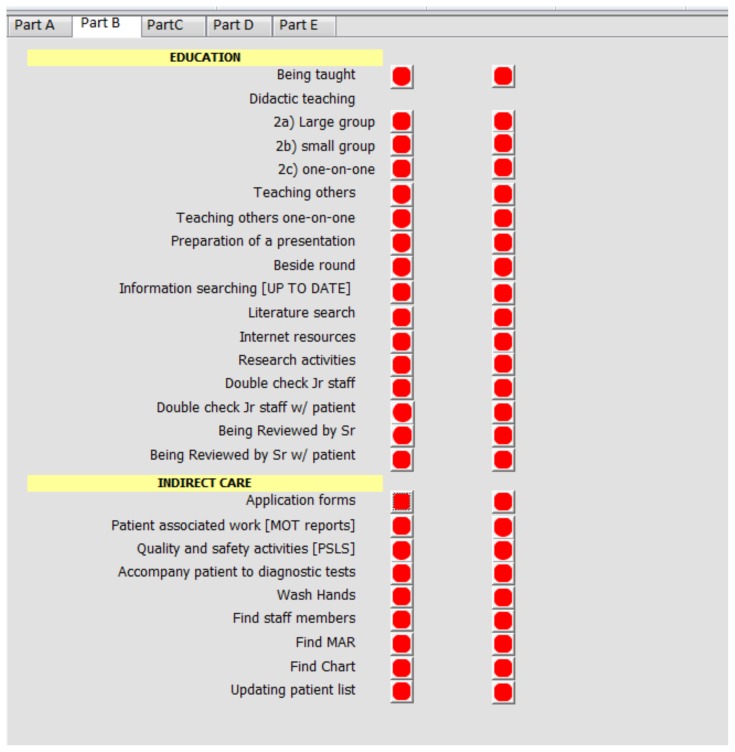
Form Layout Part B

**Figure:**
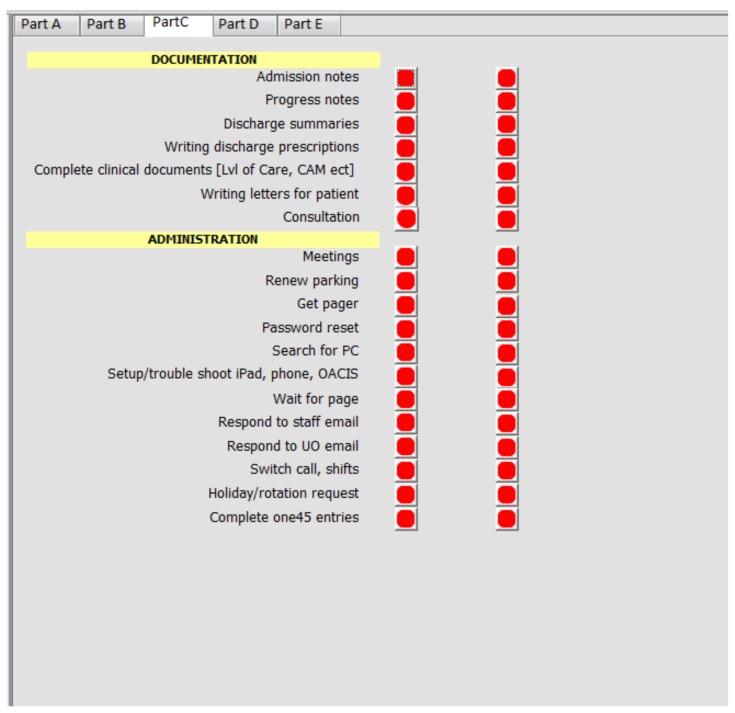
Form Layout Part C

**Figure:**
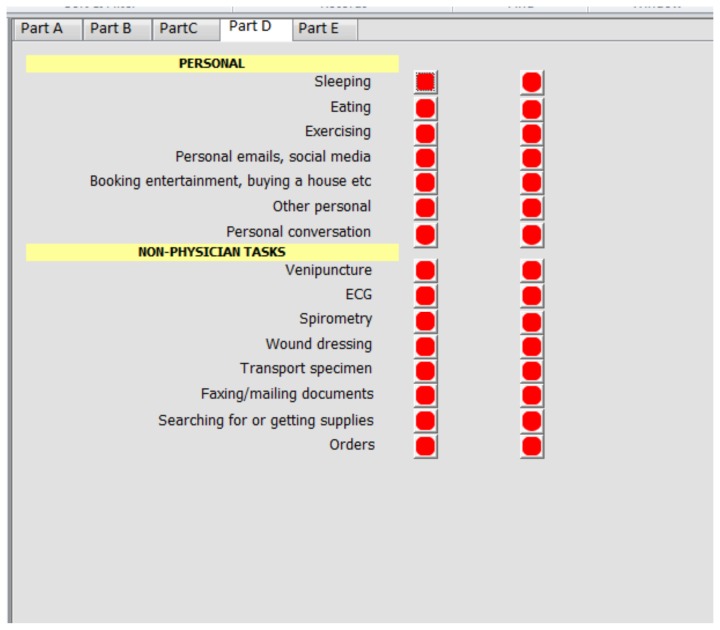
Form Layout Part D

**Figure:**
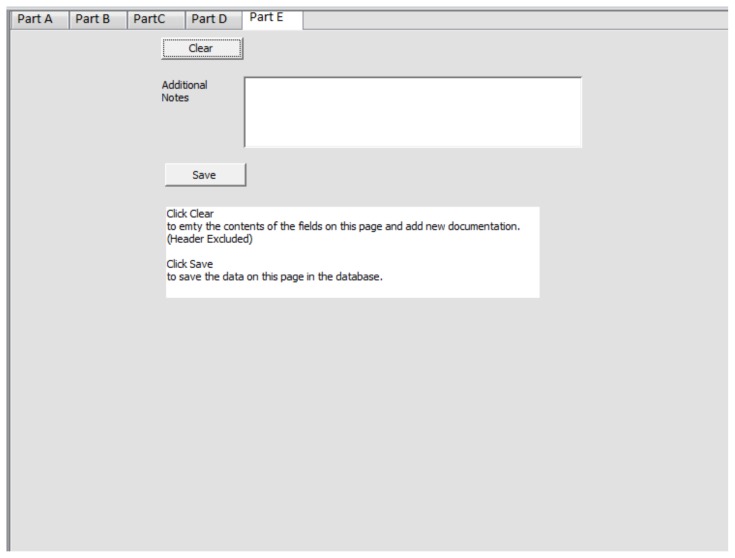
Form Layout Part E

## Appendix A List of tasks (including some clarifying comments)

**Table:**

Task category	Task description
Transit	Transit to the hospital
Transit	Travel to another hospital
Transit	Travel within hospitals
Transit	Wait for shuttle
Personal	Booking entertainment, buying a house etc
Personal	Eating
Personal	Exercising
Personal	Holiday/rotation request
Personal	Other personal
Personal	Personal conversation
Personal	Personal emails, social media
Personal	Sleeping
Direct Care	Consult CMA for results (*mobile version of EMR*)
Direct Care	Consult OACIS (*hospital EMR*)
Direct Care	CPOE orders (*electronic order entry*)
Direct Care	Doing a physical exam
Direct Care	Doing a procedure
Direct Care	Electronic orders
Direct Care	eMedRec (*electronic medication reconciliation module*)
Direct Care	Isolation procedures
Direct Care	Paper order (*writing orders on paper forms*)
Direct Care	Patient Counselling
Direct Care	Phone Orders (*transmitting orders by phone*)
Direct Care	RACE call (*Rapid Assessment of Critical Events – pre arrest team*)
Direct Care	Run Code Blue (*cardiac arrest event*)
Direct Care	Setup for procedure
Direct Care	Take history
Indirect Care	Accompany patient to diagnostic tests
Indirect Care	Application forms
Indirect Care	Find chart
Indirect Care	Reading clinical documents
Indirect Care	Find MAR (*medication administration record*)
Indirect Care	Find staff members
Indirect Care	Patient associated work
Indirect Care	Quality and safety activities
Indirect Care	Updating patient list
Indirect Care	Wash hands
Communication	Answer Page
Communication	Communication with hospital staff (team members)
Communication	Handover
Communication	Ipad consult (*reception of ER consult via mobile device*)
Communication	Phone call
Communication	Sign-in rounds
Communication	Sign-out rounds
Communication	Speaking to a staff physician
Communication	Speaking to family or friends
Communication	Text messaging send/receive
Communication	Wait for page
Documentation	Admission notes
Documentation	Complete clinical documents
Documentation	Consultation
Documentation	Discharge summaries
Documentation	Progress notes
Documentation	Writing discharge prescriptions
Documentation	Writing letters for patient
Administration	Complete one45 entries (*on-line rotation evaluation forms*)
Administration	Get pager
Administration	Meetings
Administration	Password reset
Administration	Renew parking (*permit*)
Administration	Respond to staff email
Administration	Respond to UO email (*UO=University of Ottawa*)
Administration	Search for PC (*looking for an unoccupied desktop computer*)
Administration	Setup/trouble shoot iPad, phone, OACIS (*technology help*)
Administration	Switch call, shifts
Education	Being reviewed by Sr (*case review with senior resident*)
Education	Being reviewed by Sr w/patient (*in presence of patient*)
Education	Being taught
Education	Beside round
Education	Didactic teaching:Large group (12 or *more people*)
Education	Didactic teaching:one-on-one
Education	Didactic teaching:small group (*less than 12 people*)
Education	Double check Jr staff (*case review by staff MD with junior resident*)
Education	Double check Jr staff w/patient (*in presence of patient*)
Education	Information searching
Education	Internet resources
Education	Literature search
Education	Preparation of a presentation
Education	Research activities
Education	Teaching others
Education	Teaching others one-on-one
Non-physician tasks	ECG
Non-physician tasks	Faxing/mailing documents
Non-physician tasks	Orders
Non-physician tasks	Searching for or getting supplies
Non-physician tasks	Spirometry
Non-physician tasks	Transport specimen
Non-physician tasks	Venipuncture
Non-physician tasks	Wound dressing

## Appendix B Data collection grid

**Table:**

Sunday	Monday	Tuesday	Wednesday	Thursday	Friday	Saturday	# of Shifts Observed	Total number of Observations
0300–0900	0300–0900	0300–0900	0300–0900	0300–0900	0300–0900	0300–0900	14	**N=61**
0900–1500	0900–1500	0900–1500	0900–1500	0900–1500	0900–1500	0900–1500	16	**Junior residents N=31(50.8%)**
1500–2100	1500–2100	1500–2100	1500–2100	1500–2100	1500–2100	1500–2100	14	**Senior residents N=30(49.2%)**
2100–0300	2100–0300	2100–0300	2100–0300	2100–0300	2100–0300	2100–0300	17	

## Appendix C Brief technical details of the mobile data capture application

The Time and Motion application was built on Microsoft Access 2003 and installed on a Windows Server 2003 running Citrix version 4.5 (sp 2006.10). An iPad (Apple corporation, iOS version 6.x) running a Citrix receiver application was used to remotely and securely access the database entry forms. No data resided on the iPad. The iPad used the hospital WiFi wireless network for connectivity and security and privacy were managed by password and device unique ID and network address.

There are 5 system (lookup) tables that either drove the application’s drop down lists and/or used by the Visual Basic code:

1. Time Slots (Four 6-hour windows)
2. Locations (CTU)
3. Task Categories (category in which an activity belongs)
4. Tasks Activities
5. Subjects (physician or residents)

There are 3 data entry tables as follows:

1. Main (time records every activity)
2. Main Interruptions (time records every interruption on an activity which may have 1 or more interruptions)
3. Other (additional notes that needed to be captured for a case)

There were 2 forms developed, 1 tied to the Main data entry and the second tied to each interruption. The Main form was built using the TAB feature and a total of 5 tabs were designed to capture the various tasks, each grouped under a logical tab. The Main form could only track 1 physician/resident at any one time. i.e. 1 observation, so one could not flip between different cases. Multiple users could potentially run multiple instances of the database and each could be tracking their own case. A snapshot of the Main form 1^st^ and last tab (Part A and E) are attached here. Technically, parts A, B, C, D are basically the same, i.e. other than pointing to a different activity, the logic is the same. Part E is different, it captures free text notes for that specific case. It also has the ability to capture multiple notes, hence the need for a ‘Clear’ and ‘Save’ button. By default, Access saves data as it’s entered, however this form requires an explicit ‘Save’. 1 observation/case is made up of a header (all mandatory, uses drop downs):

- Staff Physician (SP)
- Resident (Res)
- Location (CTU)
- Time Slot (TS)
- ID – auto generated number that identifies each case

Each activity in Tabs parts A, B, C, D have the same flow. Note the header (highlighted above) must be filled prior to clicking on any of the activity. Each activity has 2 buttons, the left one is the start and end time button for the activity, the right one is the interruption start and end time button for the activity. When you click the button, it will go green to indicate the time tracking for the activity has started. The right button remains red until an interruption needs to be tracked. It also turns green.

This snapshot shows an activity having started followed with an interruption which results in an additional screen to document the activity that caused the interruption. The Category and Task are the same as the ones listed in each Tab (by design). More free text details can also be provided. It is not mandatory to document anything on the Interruption screen, one can cancel out of it, however, the interruption is still tracked. The interruption must be stopped by clicking on the rightmost green button (will revert back to red) before the activity can be stopped by clicking on the leftmost green button (will revert back to red). Note the same activity can be interrupted more than once as long as the activity hasn’t been stopped.

The Main form making up the 4 TABs use the same logic for each activity with different parameters to identify each one. The sample code below is for 1 activity which is made up of 4 buttons, each tied to an Event Procedure. Consequently, there are 4 and they’re shown below. Each calls a separate function, however this same function is used for every activity. As noted, only the parameters identifying the activity (highlighted) are specific, this parameter is maintained in the Unbound field. The detailed event procedures and function calls are available on request.
